# Nature as an Ecological Asset for Positive Youth Development: Empirical Evidence From Rural Communities

**DOI:** 10.3389/fpsyg.2021.688574

**Published:** 2021-06-04

**Authors:** Edmond P. Bowers, Lincoln R. Larson, Benjamin J. Parry

**Affiliations:** ^1^Youth Development Leadership, Clemson University, Clemson, SC, United States; ^2^Department of Parks, Recreation, and Tourism Management, North Carolina State University, Raleigh, NC, United States; ^3^School of Sport, Exercise, and Rehabilitation Sciences, University of Birmingham, Birmingham, United Kingdom

**Keywords:** adolescence, connection to nature, nature, outdoor recreation, positive youth development, rural youth, psychological health

## Abstract

Despite increasing emphasis on youth contact with nature and associated benefits, research has not examined the direct relationship between adolescents' nature-based experiences and holistic measures of positive youth development (PYD). This gap may stem from the lack of interdisciplinary work on nature and PYD. Our study integrates multiple disciplinary perspectives to explore direct associations between time in nature, connection to nature, and the five Cs of PYD (competence, connection, confidence, character, and caring) as well as the “sixth” C of contribution. From 2015 to 2016, we collected data from 587 diverse South Carolina middle school students (57% female, 40% BIPOC) between the ages of 11 and 14 (*M*_age_ = 12.9) and living in predominantly low-income communities. OLS regression analyses indicated that across all youth, self-reported connection to nature was a consistent positive correlate of overall PYD and each of the individual Cs. Time in nature was associated with overall PYD and competence. Findings demonstrate significant associations between nature-based experiences and PYD and underscore the importance of ensuring that diverse youth can access and enjoy the developmental benefits of nature and nature-based recreation opportunities.

## Introduction

Evidence suggests that contemporary youth are spending less time outdoors than youth in prior generations (Kellert et al., 2017; Larson et al., 2019), and the negative consequences of this “nature-deficit disorder” for youth development may be profound (Louv, 2005; Chawla, 2015). Through engaging in the outdoors, youth often become more connected to nature (Cheng and Monroe, 2012; Rosa et al., 2019). This connection to nature can, in turn, promote happiness (Capaldi et al., 2014; Zelenski and Nisbet, 2014) and well-being (Birch et al., 2020; Jackson et al., 2021), among other beneficial cognitive, psychological, and physiological outcomes (Mayer et al., 2009; Chawla, 2015; Norwood et al., 2019). Despite increasing emphasis on benefits associated with youth exposure to nature, research has not examined the direct relationship between connection to nature and holistic measures of positive youth development (PYD). This gap is often due to the lack of interdisciplinary work on nature and PYD (Schusler and Krasny, 2010). This study is a joint effort of scholars in youth development and the recreation and conservation research disciplines to explore direct associations between time in nature, nature connectedness, and PYD, as operationalized by the five Cs of PYD and the “sixth” C of youth contribution (Lerner et al., 2015).

### Developmental Benefits Associated With Nature

Numerous studies have demonstrated the value of nature for human health across diverse populations of adults (Hartig et al., 2014; Kuo, 2015). In terms of physical health, contact with nature can promote active lifestyles that reduce the risk of cardiovascular disease and other chronic health conditions (Lachowycz and Jones, 2013; Richardson et al., 2013; Twohig-Bennett and Jones, 2018). With respect to mental health, contact with nature, whether measured directly (e.g., use of greenspace) or indirectly (e.g., proximity to greenspace), has been linked to improved cognitive functioning (Berman et al., 2008; Bratman et al., 2019), attention restoration (Kaplan, 1995), stress reduction (Hunter et al., 2019), emotional well-being (Capaldi et al., 2015), and social relationships (Weinstein et al., 2009; Jennings and Bamkole, 2019). When time in nature fuels a deeper connection to nature, often defined as an individual's “affective, experiential connection to nature” (Mayer and Frantz, 2004, p. 504), the subsequent individual health benefits (e.g., personal growth, happiness, purpose in life) and community-level benefits (e.g., pro-environmental and social justice behaviors) may be even more pronounced (Clayton and Opotow, 2003; Mayer et al., 2009; Nisbet et al., 2011; Cervinka et al., 2012; Zelenski and Nisbet, 2014; Capaldi et al., 2015; Pfattheicher et al., 2016; Bihari and Jaiswal, 2020; Martin et al., 2020; Pritchard et al., 2020). Although much remains unknown about the nature-health relationship, including the processes that promote positive health outcomes (Kondo et al., 2018), contact with the natural environment is increasingly recognized as a valuable health promotion tool (Maller et al., 2006; Van den Bosch and Sang, 2017).

The health benefits associated with nature may be particularly important for youth (Kellert, 2005; Chawla, 2015; Garst, 2018). Neighborhood greenspace is a consistent correlate of physical activity in children and adolescents (Roemmich et al., 2006, Floyd et al., 2011), and is associated with a number of other positive physical health outcomes (Maller et al., 2006; McCurdy et al., 2010; Reuben et al., 2020) including development of motor skills (Kabisch et al., 2019). Spending more time in nature through structured and unstructured outdoor activities can also improve children's mental health (Taylor et al., 2006, Taylor and Kuo, 2011; McCormick, 2017; Tillmann et al., 2018), emotional well-being (Norwood et al., 2019), and moral and psychosocial development (Kellert, 2005; Ginsburg, 2007; Dowdell et al., 2011; McCormick, 2017). Youth exposure to nature can boost creativity (Chawla, 2015; Williams et al., 2018) and may help to enhance students' academic performance (Browning and Rigolon, 2019). Time in nature has been identified as a buffer for stress in both urban (Corraliza et al., 2012) and rural (Wells and Evans, 2003) children, even in formal education settings (Chawla et al., 2014). Nature-based experiences can also bolster resilience, helping maintain emotional well-being when confronted with traumatic events (Touloumakos and Barrable, 2020), including the COVID-19 pandemic (Jackson et al., 2021). When increased contact with nature fuels a stronger connection to nature, the cognitive, affective, and physical benefits youth derive from nature are likely to increase (Cheng and Monroe, 2012; Barrera-Hernández et al., 2020). For all of these reasons, as the diverse health benefits of nature for youth become more apparent, physicians are beginning to prescribe exposure to nature for children experiencing physical and mental health issues (Seltenrich, 2015; Kondo et al., 2020).

Despite the wealth of research examining the health benefits associated with youth time in nature and connection to nature, few studies have specifically examined the salutogenic value of nature-based experiences through the lens of strength-based approaches to adolescence such as the PYD perspective (Mainella et al., 2011; Chawla, 2015). This gap reflects the lack of interdisciplinary work on nature and PYD (Schusler and Krasny, 2010), where inferences drawn from the public health, environmental psychology, and other fields of research referenced above are rarely integrated with insights from child psychology and developmental science (Parry et al., 2021a). Research and practice focused on the developmental benefits derived from nature might therefore be enhanced through a PYD approach (Passarelli et al., 2010).

### The PYD Perspective

The PYD perspective arose in the 1990s as interests in the strengths of youth, the relative plasticity of human development, and resilience came together to foster the development of the concept of PYD (Lerner et al., 2009, 2015). Application of the PYD perspective as a frame for research and practice has grown exponentially as it is at the forefront of contemporary approaches to adolescence (Lerner et al., in press). Models of PYD are based on relational developmental systems (RDS) metatheory of human development (Lerner et al., 2015; Overton, 2015). The conceptual emphasis in RDS-based theories is placed on mutually influential bidirectional relations between individuals and contexts. There are several models of PYD based on RDS ideas (e.g., see Lerner et al., 2015, for a review). The most empirically supported of these PYD models is Lerner and Lerner's Five Cs model (Heck and Subramaniam, 2009; Lerner et al., 2015).

In the Five Cs model, when individual youth strengths (e.g., intentional self-regulation, hopeful future expectations) are aligned with resources from key contexts of their lives (e.g., families, schools, or communities), youth are more likely to thrive as marked by the five Cs of PYD (competence, confidence, character, caring, and connection; Bowers et al., 2010; Geldhof et al., 2015). Competence refers to a young person's ability to successfully navigate the complex environments within which they live. Confidence is an internal sense of overall positive self-worth and self-efficacy. Character includes respect for social norms, engagement in prosocial behavior, and knowledge of right and wrong. Connection are the positive bonds with others that youth possess in their lives; however, an important component of connection is the sense of value and belonging that youth feel because of their relationships with others. Caring refers to a youth's sense of compassion, sympathy, and empathy for others. In turn, youth exhibiting the five Cs will be more likely to contribute to their families, schools, and communities, with contribution often referred to as the “sixth” C (Lerner, 2004; Agans et al., 2014; Geldhof et al., 2015; Lerner et al., 2015). That is, thriving youth tend to possess an other-oriented ideology and act in ways that enhance their families, schools, and communities, and possibly the natural environment in which they exist (Tidball and Krasny, 2010). Youth contribute to these settings in diverse ways, from helping parents at home, participating in student government, organizing community clean-ups, and volunteering to engaging in civically-oriented actions such as protesting and activism (Zaff et al., 2010; Hershberg et al., 2015).

Although research has shown the many ways PYD outcomes are impacted by resources associated with families, schools, afterschool programs, and other youth settings (Bowers et al., 2015), little research has explored the multiple ways that nature can serve as a context which provides resources to promote PYD (i.e., ecological developmental assets; Benson et al., 2006, 2011). Ecological developmental assets are “environmental, contextual, and relational features of socializing systems” that serve as “developmental nutrients” for positive outcomes in diverse youth (Benson et al., 2011, p. 198). Structured outdoor experiences, such as camps and adventure education, may provide youth with unique opportunities for growth and development in areas such as self-concept, resiliency, interpersonal skills, problem solving, and leadership (Sibthorp et al., 2007; Garst et al., 2011; Bowers et al., 2019). For example, reviews suggest that adolescents who attend adventure programming report PYD outcomes that are 62–65% higher than their peers (Hattie et al., 1997). More recently, qualitative evidence suggests outdoor-based programs can be an effective approach to promoting the five Cs (Mercier et al., 2019; Parry et al., in press). In many cases, structured outdoor experiences contain key elements such as youth-adult mentoring, positive peer connections, and empowering activity engagement that fuel PYD in other settings (Vandell, 2013; Bowers et al., 2019). Less formal nature-based experiences may play an important role as well. Research has increasingly shown that unstructured outdoor play is a powerful precursor to PYD (Mainella et al., 2011; Milteer et al., 2012), producing many of the physical, mental, and psychosocial health outcomes described above. Even indirect exposure to nature, often measured via access and close proximity to greenspaces, has been linked to positive mental and physical health outcomes in children (Maas et al., 2006; McCormick, 2017; Dzhambov et al., 2018). By providing opportunities for authentic engagement (i.e., time in nature) and spiritual connection (i.e., connection to nature), nature could be a key ecological asset contributing to place-based youth development (Benson and Saito, 2001; Shek et al., 2019).

### Contributions of the Current Study

Our study addresses several gaps that exist in the current literature on the developmental benefits associated with nature. First, most studies examining the links between nature, greenspace, and healthy outcomes have been conducted with adult samples. Youth may experience and connect to nature in different ways than adults, and the processes linking nature-based experiences to outcomes may function differently (Chawla, 2015). In addition, much of the existing work on nature-related health benefits for youth has also largely been aimed at preventing or reducing negative outcomes in youth. Although these studies contribute to the evidence base supporting the benefits of nature for youth, work from a strengths-based PYD perspective can complement and extend this work by providing evidence that nature might serve as a contextual resource to promote positive youth developmental outcomes in a more holistic manner (Dustin et al., 2009). That is, many prior studies focus on singular mental or physical health outcomes or a small set of outcomes within cognitive, social, emotional, or physical domains of development. Exploring the links between youth experiences of nature and a comprehensive measure of youth development, such as the Five Cs model (Lerner et al., 2015), can help to clarify the multifaceted developmental benefits of nature.

Finally, few studies of youth outdoor time and connection to nature have focused on rural contexts. For example, positive relationships between green space and children's physical activity have been identified in urban communities (Roemmich et al., 2006; Boone-Heinonen et al., 2010); however, analogous research on the benefits of green space on youth in rural areas is lacking (Michimi and Wimberly, 2012; Larson et al., 2015). Rural youth's experiences and understanding of nature may differ from those of children from cities (King and Church, 2013; Kellert et al., 2017), and rural youth may have more opportunities to engage with nature than their urban peers (Sandercock et al., 2014; Matz et al., 2015). Reflective of RDS models, experiences within rural youth may also be heterogeneous across different demographic groups, with certain groups such as females and African Americans less likely to spend time in nature (Larson et al., 2019). As with research on connection to nature, most work based on the Five Cs model of PYD has also overlooked rural populations of youth. Much of the evidence base for the Five Cs model has been derived from the 4-H Study of PYD (Lerner et al., 2005), which is marked by limited racial, ethnic, socioeconomic, and place variability (Bowers et al., 2014; Spencer and Spencer, 2014). The present study broadens the literature exploring nature and PYD in diverse groups of young people by focusing specifically on rural youth.

Our study sought to integrate disciplines and explore, through the lens of PYD, the capacity of nature to promote healthy and positive development in young people. We aimed to answer one primary research question: Is there a relationship between time in nature, connection to nature, and PYD outcomes? We did this by examining associations between different measures of nature-based experience and connection and the five Cs of PYD plus contribution, while controlling for demographic attributes within a diverse sample of adolescents living in rural South Carolina, USA.

## Materials and Methods

From 2015–2016 we surveyed middle school students in rural areas across the state of South Carolina, USA, as part of a larger study on the antecedents and outcomes of PYD in youth living in low-income, rural communities. There are several reasons why South Carolina is an ideal context for PYD research. In 2016, 23% of children under age 18 in South Carolina were living in poverty, with 12.7% of children living in areas of concentrated poverty (Annie E. Casey Foundation, 2018). In 2016, South Carolina ranked 34th out of 50 U.S. states in terms of economic well-being; in terms of overall child well-being, South Carolina ranked 38th out of 50 states (Annie E. Casey Foundation, 2018). These concerns are exacerbated in rural areas of the state. According to the U.S. Census' Urban and Rural Areas definition (Ratcliffe et al., 2016), 34% of the South Carolina population is considered “rural,” with the poverty rate in rural South Carolina at 20.6% as compared to 12.8% in urban areas of the state (United States Census Bureau, 2020).

Based on U.S. Census data, we systematically selected middle schools and out-of-school program sites (e.g., 4-H organizations) in these rural areas that were located in low-income communities with racially and ethnically diverse populations. A total of 700 students at 18 different sites completed the questionnaire, with the number of surveys completed at each site ranging from 14 to 132 and response rates ranging from 13.4 to 100% (overall response rate = 37.9%). To ensure that all students in the sample were indeed middle school-aged youth from rural areas, we filtered out any respondents not in grades 6–8 and those from school districts coded as “city” or “suburb” based on the NCES Locale Classifications and Criteria. This resulted in an effective sample size of 587.

### Sample

All youth in our sample of 587 students were either in 6th (15%), 7th (45%), or 8th grade (40%). Over half of the sample was female (57%) and identified as white (60%), with other racial/ethnic representation including African Americans (25%), Hispanic/Latinos (5%), and Other races (10%). Ages of youth participants ranged from 11 to 14 (M = 12.9 years, SD = 0.73). School data were available for 92% of participants, revealing that most students in the sample (77%) attended Title I schools (i.e., schools with high percentages of children from low-income families).

### Measures

#### Time in Nature and Connection to Nature

We measured youth-reported time in nature using one item. The item focused on nature-based outdoor time by asking youth, “In the past week, including Saturday and Sunday, about how many *hours per day* did you spend *outdoors in nature* (in a park, a forest, a backyard or school playground with trees, or similar place)?” The question incorporated time spent in nature during school and outside of the school setting (i.e., during leisure time), and youth were encouraged to provide their best estimate of average time use across both weekdays and weekend days. By providing a list of possible activity settings, the question helped to clarify the broad definition of “nature” (Larson et al., 2019). Response options included the following categories, with assigned values for data analysis based on the midpoint of the range for each response option: none (0 h), <0.5 h per day (0.25 h), between 0.5 and 1 h per day (0.75 hr), between 1 and 2 h per day (1.5 h), between 2 and 3 h per day (2.5 h), between 3 and 4 h per day (3.5 h), between 4 and 5 h per day (4.5 h), >5 h per day (5.5 h). Although responses to this self-reported, single-item metric may be affected by recall bias or inaccurate characterization of discretionary time, similar data collection strategies has been effectively employed and interpreted in a variety of other studies examining youth time outdoors (Larson et al., 2011; Outdoor Foundation, 2018).

We measured youth connection to nature with an adapted version of a measure originally designed for adults, a 7-item short form measure of the *Nature Relatedness* (NR) scale created by Nisbet et al. (2009) and Nisbet and Zelenski (2013). The NR scale includes items from two dimensions: NR-experience (physical familiarity and comfort with the natural world) and NR-self (personal connection to and internalized identification with nature). Employing the version of the NR short form scale that Larson et al. (2019) adapted for use with adolescents, we used three items to measure both NR-experience (Cronbach's α = 0.87, McDonald's ω = 0.87) and NR-self (Cronbach's α = 0.83, McDonald's ω = 0.83; Supplementary Table 1). An example item for NR-experience was “My favorite places are outside in nature.” An example item for NR-self was “I feel very connected to all living things and the Earth.” Responses ranged from *1* = *Strongly disagree* to *5* = *Strongly agree*. A composite score for each dimension was calculated by averaging across the items.

**Figure 1:**
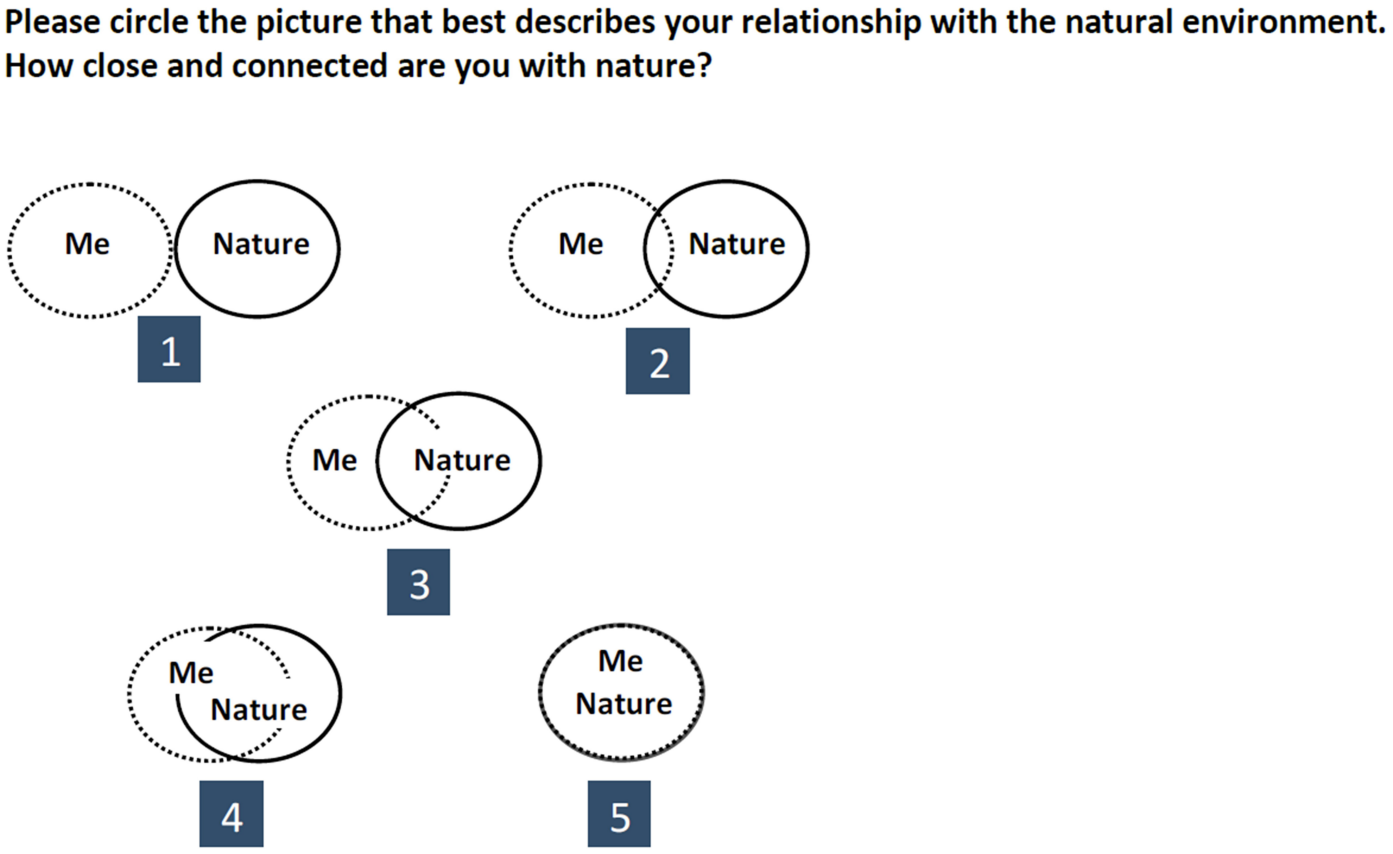
Single item used to measure youth connection with nature, adapted from the Inclusion of Nature in Self (INS) scale developed by Schultz (2001).

We also used the *Inclusion of Nature in Self* (INS) scale created by Schultz (2001) to measure youth connection to nature. The INS is a single-item metric that measures the perceived relationship or interaction between the self and nature (Figure 1). It provides a parsimonious and straightforward measurement approach due to its graphical design. Despite its simplicity, this scale is correlated with other measures of nature connectedness, commitment, and identity (Lieflander et al., 2013). The INS test–retest correlations have also provided very high reliabilities between measurement times with a retest given 1 or 4 weeks after the initial test (Schultz et al., 2004), and the scale is increasingly being used with diverse audiences, including youth (Kleespies et al., 2021). We converted student responses on the INS scale to into a single integer score ranging from *1* = *No connection* to *5* = *Complete connection*.

#### Positive Youth Development: Five Cs and Contribution

Positive youth development (PYD) was assessed using the adapted version of 34-item Short Form measure of the Five Cs of PYD (PYD-SF; Geldhof et al., 2014). The 34 items reflected the five Cs: competence, confidence, character, caring, and connection (Supplementary Table 2). Competence was measured by six items indexing academic, social, and physical competence (e.g., “I am just as smart as others my age.”). Confidence was measured by six items indexing self-worth, positive identity, and appearance (e.g., “I am happy with myself most of the time.”). Character was measured using eight items indexing social conscience, valuing diversity, conduct morality, and personal values (e.g., “It is important to me that I help make the world a better place to live in.”). Caring was measured by six items indexing sympathy and empathy (e.g., “When I see someone being taken advantage of, I want to help them.”). Finally, connection was measured by eight items indexing positive bonds with family, neighborhood, school, and peers (e.g., “In my family I feel useful and important.”). An average score across items was calculated for each dimension, with higher scores reflecting higher levels of positive youth development. A global PYD score was also calculated by averaging scores across all 34 items. Both the global PYD scale (Cronbach's α = 0.88, McDonald's ω = 0.87), and the specific subscales measuring each dimension of PYD - competence (α = 0.74, ω = 0.76), confidence (α= 0.84, ω = 0.83), character (α = 0.65, ω = 0.65), caring (α = 0.83, ω = 0.84), and connection (α = 0.80, ω = 0.81) - demonstrated acceptable psychometric properties. All PYD items used in the study are reported in Supplementary Table 2, and full details about these measures, their construction, and validity and reliability can be found in Geldhof et al. (2014).

To create a composite score for the “sixth” C, contribution, participants responded to twelve items which were weighted and summed to measure contribution (Supplementary Table 3). These items were from four subsets: leadership, service, helping, and ideology. Items from the leadership, service, and helping scales measured the frequency of time youth spent helping others (e.g., friends or neighbors), providing service to their communities, and acting in leadership roles. Together, the leadership, service, and helping subsets comprise an action component of contribution. The ideology scale measured the extent to which contribution was an important facet of their identities (e.g., “It is important to me to contribute to my community and society”). These items are derived from existing instruments with known psychometric properties and used in large-scales studies of adolescents, including the Profiles of Student Life-Attitudes and Behaviors Survey (PSL-AB; Benson et al., 1998) and the Teen Assessment Project Survey Question Bank (TAP; Small and Rodgers, 1995). The action and ideology components were weighted equally to calculate the contribution composite scale score, which demonstrated acceptable psychometric properties (Cronbach's α = 0.73, McDonald's ω = 0.71; Supplementary Table 3) The reliability estimates for the current sample are consistent with prior work on the five Cs and contribution (e.g., Geldhof et al., 2014).

### Procedure

The survey instrument was completed in either paper format or through an online Qualtrics survey (Qualtrics, Provo, UT) with trained study staff on hand. In both modalities, youth completed the survey in a group setting with peers present and submitted the survey to the study staff or through the online portal once they were finished. All surveys were completed outside of instructional time (in schools) or outside of activity time (in out-of-school programs). We attempted to limit the potential for response bias using several empirically supported strategies (Bowling, 2005). First, the study staff received training on the protection of human subjects; they were also former teachers familiar with youth settings. Second, the study staff provided assurances of anonymity to participants. Finally, appropriate classroom management techniques were implemented during the administration of the surveys so that youth completed the surveys on their own; talking among youth during survey administration was not permitted.

### Data Analyses

Prior to analyses, we assessed the psychometric properties of items on the preexisting and previously validated connection to nature and PYD scales. To confirm scale reliability and internal consistency, we used Cronbach's alpha (α; Vaske, 2008) and McDonald's omega (ω; Hayes and Coutts, 2020). After exploring descriptive statistics and frequencies associated with each variable, we examined bivariate correlations between all variables of interest. We treated all PYD scale means as continuous variables, an acceptable practice when response scales contain five or more categories (Rhemtulla et al., 2012). This enabled us to conduct a series of OLS regression models to investigate the relationship between PYD indicators, time in nature, and connection to nature, controlling for participants' demographic attributes. We ran seven separate models where the dependent variables were the means of the global PYD scale, the five Cs of PYD, and contribution. We tested to ensure all modeling assumptions, including approximately normal distribution the absence of multicollinearity (VIF <2.0), were satisfied before proceeding. Model fit was assessed using Adjusted *R*^2^. We used parameter estimates and standardized parameter estimates (with estimated 95% confidence intervals) to assess the strength of association between time in nature, connection to nature, demographic attributes, and the different dimensions of PYD. To minimize the risk of Type II (i.e., false negative) errors in our exploratory analysis, we used a cutoff criterion for statistical significance of α = 0.10 or lower (Mudge et al., 2012). All data were analyzed using IBM SPSS Statistics, Version 25.0 (IBM Corp., 2019).

## Results

Youth in our sample reported moderate levels of time in nature and connection to nature. The average amount of time youth reported spending outdoors in nature during a typical day was 1.71 h (SD = 1.57), with 51.3% reporting spending at least 1 h outdoors daily. On the nature relatedness scales, NR-experience scores (*M* = 3.93, *SD* = 1.00, 59.1% reporting 4.0 or higher out of 5) were slightly higher than NR-self scores (*M* = 3.53, *SD* = 1.00, 39.3%), though both means were well above the scale midpoint. The mean score for INS scale was 3.40 (*SD* = 1.31), with 49.1% of youth reporting scores of 4.0 (“very connected”) or higher.

Adolescents in our sample scored relatively high on most PYD metrics, including the global PYD scale (*M* = 4.01, *SD* = 0.43), with 56.1% of youth scoring 4.0 (out of five) or higher. Mean scores for PYD sub-dimensions were, in descending order: caring (*M* = 4.34, *SD* = 0.73, 78.3% scoring 4.0 or higher), confidence (*M* = 4.25, *SD* = 0.64, 74.7%), connection (*M* = 3.97, *SD* = 0.66, 56.1%), competence (*M* = 3.81, *SD* = 0.66, 46.7%), and character (*M* = 3.79, *SD* = 0.56, 44.4%). Contribution scores were slightly above the scale midpoint (*M* = 59.76, *SD* = 15.21), with 73.0% of youth scoring 50 or higher (out of 100).

Bivariate correlation analyses revealed significant positive relationships between the various dimensions of PYD and our measures of both time in nature and connection to nature (Table 1). As expected within the antecedent and outcome variable groups, all nature variables and all PYD variables were also correlated with each other. Among measures of nature-based experiences, connection to nature scales were more strongly correlated with each other than with time spent outdoors.

**Table 1:** Bivariate correlations depicting relationships among time in nature, connection to nature, and overall positive youth development (PYD) scale scores in a sample of rural middle school students in South Carolina, USA (*n* = 587).

	**Time out**	**NR-Exp**	**NR-Self**	**INS**	**PYD-Full**	**Comp**.	**Conf**.	**Char**.	**Conn**.	**Car**.	**Cont**.
Time Outdoors	1.000										
NR-Experience	0.469	1.000									
NR-Self	0.333	0.584	1.000								
INS	0.474	0.608	0.588	1.000							
PYD-Global	0.194	0.328	0.418	0.331	1.000						
Competence	0.286	0.332	0.231	0.309	0.658	1.000					
Confidence	0.121	0.153	0.187	0.146	0.676	0.531	1.000				
Character	0.082	0.169	0.347	0.208	0.678	0.212	0.253	1.000			
Connection	0.154	0.242	0.283	0.280	0.729	0.417	0.437	0.276	1.000		
Caring	0.021	0.207	0.345	0.168	0.586	0.110	0.117	0.520	0.520	1.000	
Contribution	0.096	0.244	0.375	0.302	0.567	0.344	0.275	0.443	0.443	0.378	1.000

*All shaded cells are statistically significant at α = 0.05. Darker shades represent stronger associations. Correlations among nature variables depicted in green. Correlations among PYD variables are depicted in orange. Correlation between nature and PYD variables are depicted in blue*.

*NR-Exp, Nature Relatedness-experience Scale (Nisbet et al., 2009)*.

*NR-Self, Nature Relatedness-self Scale (Nisbet et al., 2009)*.

*INS, Inclusion of Nature in Self scale (Schultz, 2001)*.

Time in nature was most strongly related to youth competence (*r* = 0.286, *p* < 0.01). NR-experiences scores were also most strongly related to competence (*r* = 0.332, *p* < 0.01) but were also associated with global PYD (*r* = 0.328, *p* < 0.01). NR-self and INS scores were also most strongly related to global PYD (*r* = 0.418 and *r* = 0.331, respectively, *p* < 0.001). In general, when considering the measures of nature experiences and connection, NR-self scores were the strongest and most consistent correlate of the Cs, exhibiting the highest correlations to PYD-Global and five of the six Cs.

**Figure 2:**
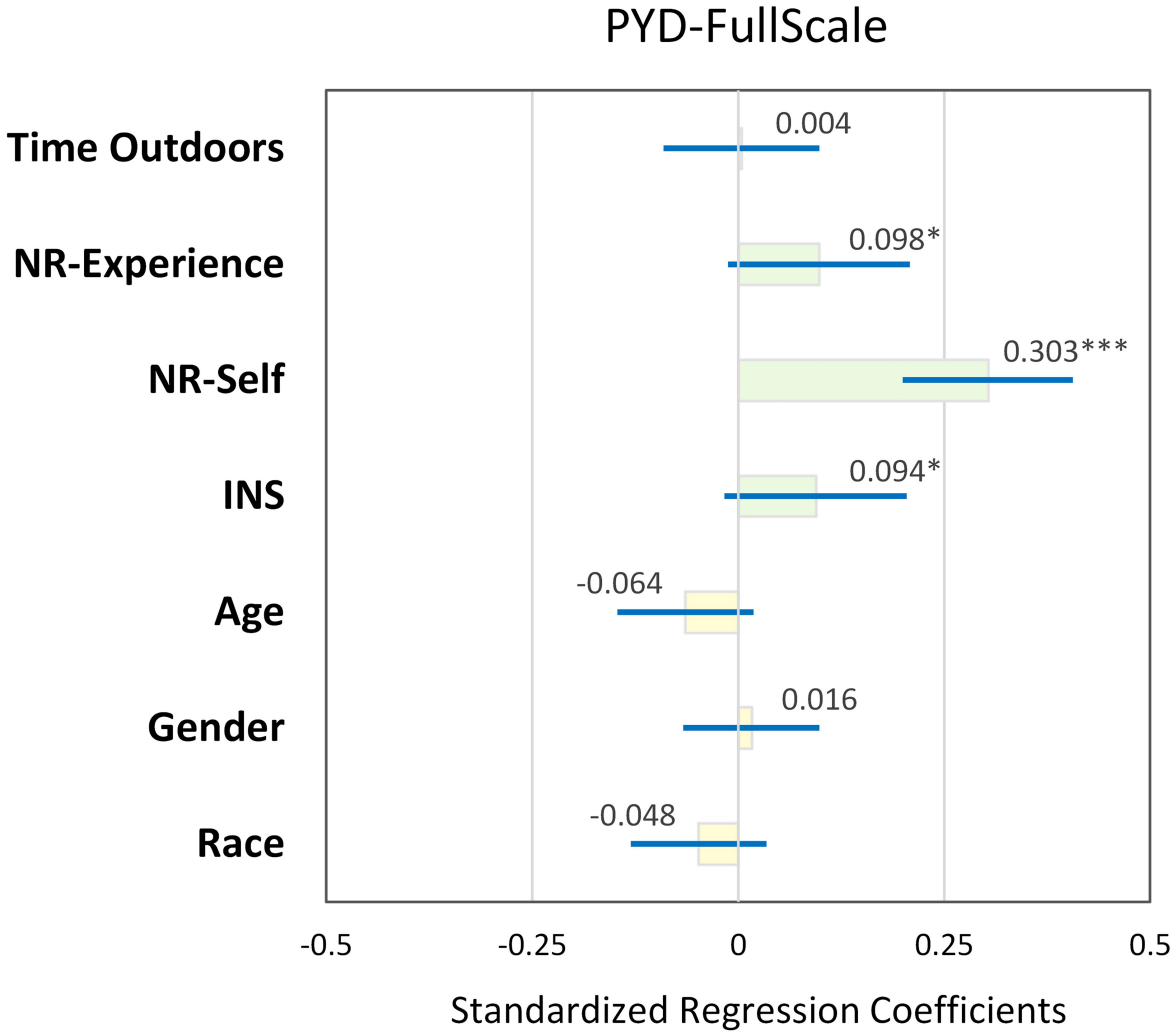
Standardized coefficients (β, with 95% CI) in OLS regression model examining association between time in nature (Time Outdoors), connection to nature [Nature-relatedness: Experience, Nature-relatedness: Self, Inclusion of Nature in Self (INS) Scale], and overall positive youth development (PYD) scale scores in a sample of rural middle school students in South Carolina, USA, controlling for demographic variables (*n* = 587). *, **, and *** indicate significance of standardized β at α = 0.10, 0.05, and 0.01, respectively.

On average, our OLS regression models examining multivariate relationships between time in nature, connection to nature, and PYD yielded moderate predictive power (Adjusted *R*^2^ values ranged from 0.074 to 0.189). We found that NR-self, NR-experience, and INS scores were associated with global PYD scale scores, controlling for demographic variables (standardized βs ranged from 0.09 to 0.30, *p* < 0.10; Figure 2, see Supplementary Table 4 for details).

**Figure 3:**
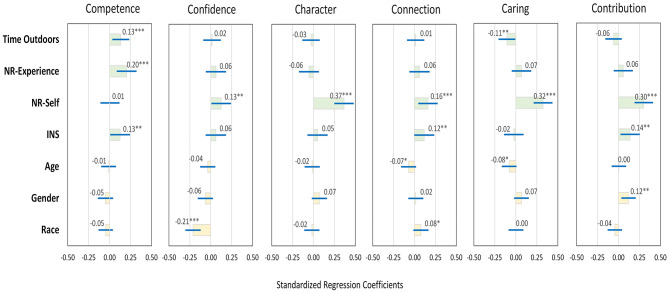
Standardized coefficients (β, with 95% CI) in OLS regression models examining associations between time in nature (Time Outdoors), connection to nature [Nature-relatedness: Experience, Nature-relatedness: Self, Inclusion of Nature in Self (INS) Scale], and different dimensions of positive youth development (PYD 5 Cs, plus contribution) in a sample of rural middle school students in South Carolina, USA, controlling for demographic variables (*n* = 587). *, **, and *** indicate significance of standardized β at α = 0.10, 0.05, and 0.01, respectively.

NR-self was positively associated with all but one of the dimensions of PYD, including contribution, caring, character, connection, and confidence (standardized βs ranged from 0.13 to 0.37, *p* < 0.05; Figure 3, see Supplementary Table 5 for details). The INS scale correlated with connection, contribution, and competence (βs ranged from 0.12 to 0.14, *p* < 0.05). NR-experience and time outdoors were positively linked to only one sub-dimension: competence (β = 0.20 and β = 0.13, respectively, both *p*s < 0.01). Time outdoors was negatively associated with caring (β = −0.11, *p* < 0.05). We also observed significant associations with demographic control variables: female students scored higher for contribution (β = 0.12, *p* < 0.05), white students scored higher for connection (β = 0.08, *p* < 0.10) and lower for confidence (β = −0.21, *p* < 0.01), and older students reported lower scores for caring and connection (β = −0.07 and β = −0.08, respectively, *p* < 0.10; Figure 3, see Supplementary Table 5 for details).

## Discussion

Growing evidence indicates that youth are spending less time outdoors (Larson et al., 2019), and this deficiency is linked to detrimental effects on health and well-being (Louv, 2005; Chawla, 2015). Given that nature is an understudied source of ecological assets (Garst, 2018), the present study viewed youth time in nature and connection to nature from a strengths-based, PYD perspective to examine the links between these “natural” ecological assets and the six Cs of PYD (the five Cs, plus contribution). We found that adolescents in rural South Carolina communities reported moderate to high levels of time in nature and connection to nature, and relatively high scores on most of the Cs. After controlling for demographic variables, results indicated that exposure and connection to nature were significantly and positively associated with PYD outcomes, with models explaining 7.4–16.2% of the variance in individual Cs and accounting for 18.9% of global PYD scores. Connection to nature, which we measured using the NR-self and INS scales (Nisbet and Zelenski, 2013; Larson et al., 2019), appears to be a particularly robust correlate of many dimensions of PYD. These findings highlight the important and potentially transformative role that nature plays as a developmental resource for diverse youth (Garst, 2018; Charles and Louv, 2020) and are consistent with prior empirical evidence pointing to the benefits of nature for physical, cognitive, social, and emotional outcomes in young people (e.g., Ginsburg, 2007; McCormick, 2017; Norwood et al., 2019; Reuben et al., 2020). The present findings, however, extend this body of work as they indicate the multifaceted benefits of time in nature and connection to nature using a comprehensive measure of healthy and positive youth development, the six Cs.

Youth who reported greater personal connection to and internalized identification with nature also reported greater levels of healthy and positive relationships in their families, peer groups, schools, and communities (i.e., the C of connection). Healthy connections across social contexts are strongly related (e.g., Bowers et al., 2010). Perhaps connection to nature might be viewed as a context-specific conceptualization of connection. Alternately, the associations between connection to nature and the C of connection could also reflect prior evidence that experiences in the outdoors are linked to positive social relationships and opportunities to build social capital (Weinstein et al., 2009; Jennings and Bamkole, 2019; Beery et al., 2020). For example, in a review of immersive nature experiences, findings suggest social health benefits are reflected through feelings of social support, connectedness to peers, and social skill development (Mygind et al., 2019).

Positive associations between connection to nature, character, caring, and contribution may reflect associations between connection with nature, environmental responsibility (Bihari and Jaiswal, 2020), and pro-environmental behavior (Whitburn et al., 2020). Through the lens of connection to nature, character reflects young people's respect for the environment and caring implies adopting prosocial norms to protect it. With an increasing number of young people being more mindful of their environmental impact, there is a shift in cultural norms toward taking more environmental responsibility (Sachs et al., 2020). As such, the C of character, which centers on respect for societal and cultural values, could be harnessed to reflect stronger pro-environmental values and morality (Pfattheicher et al., 2016). In a similar way, associations between connection to nature and caring reflect a common concern for the world in which one lives, fueled by empathy (Di Fabio and Kenny, 2021). Indeed, recent work has indicated that youth who show higher levels of compassion for their peers may demonstrate greater capacity for dispositional environmental empathy and, subsequently, stronger connection to nature and pro-environmental tendencies (Pfattheicher et al., 2016; Brown et al., 2019). Additionally, nature-based recreation strengthens sense of place, which leads to greater community involvement (Larson et al., 2018). As a young person becomes more attached to a place through engagement in nature, they may develop a stronger sense of community and, in turn, become more socially and civically engaged to contribute to their communities (Kudryavtsev et al., 2012; Zelenski et al., 2015; Flanagan et al., 2019). These relationships demonstrate how youth engagement in nature can enhance both individual (Wray-Lake et al., 2019) and community health and well-being (Tidball and Krasny, 2010).

Nature-based experiences were also linked to overall PYD, but the effect of direct experience and time outdoors was most strongly associated with competence. Research suggests that both structured outdoor recreation experiences such as camps (Garst et al., 2011; Bowers et al., 2019) and unstructured outdoor play (Mainella et al., 2011) can foster self-concept and skill development in youth, fueling sense of competence. Prior work has suggested that improvements in outcomes such as self-efficacy (akin to competence in the Five Cs model), may be mediated by engagement in nature above and beyond connection with nature (for a review, see Mygind et al., 2019). Without breaking competence down into more specific domains (i.e., physical, social, academic), it is difficult to know how and why connection to nature was positively associated with this construct. However, previous research has pointed to the benefits of time in nature for motor skill development (Kabisch et al., 2019) and academic achievement (Browning and Rigolon, 2019). In addition, competence is also pertinent when considering how people engage with nature. Roczen et al. (2014) suggest pro-environmental competence is a composite of “intellectual and motivational aptitudes that ultimately advance a person's propensity to act in an ecological manner” (p. 973). Core drivers in adolescents' environmental competence are attitudes toward and connection with nature, which in turn predict ecologically conscious behaviors (Roczen et al., 2014; Otto and Pensini, 2017). Furthermore, experiences with nature from a young age are likely to shape environmental competence and capacity to engage in pro-environmental behavior into adulthood (Rosa et al., 2018). In short, outdoor experiences and connection to nature during adolescence can help build competence and confidence that lasts a lifetime (Bialeschki et al., 2007).

### Future Research on PYD and Nature

Future research could build on this study in several ways. First, our data were collected using previously validated scales through self-report by participants. This design introduces concerns for social desirability bias, common method variance, and recall issues, especially with respect to nature-based activities. Additional measures of how youth spend their time in nature would strengthen our ability to understand links between nature and PYD. For example, future research could employ more objective and precise measures incorporating different dimensions of outdoor time and contact with nature collected via youth time diaries (Hofferth and Sandberg, 2001; Rideout et al., 2010), GPS tracking of activity patterns (Cooper et al., 2010), or other strategies (Holland et al., 2021). Expanded measurement strategies might also help to explain the unexpected link between time in nature and lower caring scores. For example, whether time outdoors is solitary vs. social can impact connection to nature in different ways (Szczytko et al., 2020), and whether nature-based experiences are structured or unstructured moderates their relation to PYD and mental health outcomes (Tillmann et al., 2018; Mygind et al., 2019). Although the reliability of PYD measures within our sample was acceptable (Hayes and Coutts, 2020), Cronbach's α and McDonald's ω values were relatively low for the character and contribution scales. These values are consistent with prior work on the Cs of PYD (Geldhof et al., 2014). However, contemporary approaches to PYD have questioned whether existing measures of thriving are valid indices across diverse populations of youth (Geldhof et al., 2015). Recent work on culturally and contextually relevant measures of PYD has particularly focused on measures of character (Lerner et al., 2017) and contribution (Hope and Jagers, 2014; Hershberg et al., 2015). Additional research should not only continue to investigate the psychometric properties of these PYD scales and their predictive validity (Hunsley and Meyer, 2003), but also explore their relevance across diverse populations including youth from rural communities.

Our study was cross-sectional, and it was not possible to determine the directionality of the associations between the constructs of interest. For example, although we frame time in nature and connection to nature as contextual resources predicting PYD, youth exhibiting high levels of the Cs may be more competent and confident to explore nature and engage in outdoor activities. As PYD models posit that processes of human development may be non-recursive (Lerner et al., 2015), future longitudinal work, perhaps using more sophisticated analytical techniques such as structural equation modeling, could examine mechanistic pathways and processes through which nature experiences and connection to nature foster PYD (Bratman et al., 2012; Kuo, 2015). As PYD models are derived from relational developmental systems (RDS) metamodels and posit that youth thriving is best promoted when individual youth strengths are aligned with resources in their contexts, future studies on nature and PYD could include youth strengths such as intentional self-regulation, hope, and spirituality. These strengths have been linked to the five Cs of PYD, and often moderate the effect of contextual resources on the five Cs (e.g., Bowers et al., in press).

Future PYD-based approaches should also account for the multiple systems within which youth are embedded. For example, parents and caregivers are important to consider when exploring associations between nature and PYD. Prior research has indicated that parents serve as role models for engagement with nature (Larson et al., 2013; McFarland et al., 2014) and parental recognition of the benefits of nature is key predictor of youth outcomes (Larson et al., 2013). An RDS approach examining effects of nature on PYD could also account for diverse opportunities for youth engagement with nature by documenting the multiple activities in which youth participate (Sanders et al., 2015) and tracking the “greenness” of school and community environments, which may also influence cognitive and psychological outcomes (Chawla et al., 2014).

When considering young people's broader ecology, there is also a need to explore the role of connection to nature in urban communities. Our study focused on youth living in rural communities within a southeastern US state. Rural youth often experience greater access to nature and higher levels of participation in nature-based recreation activities that urban or suburban youth (Kellert et al., 2017). Thus, the relatively high levels of PYD outcomes observed in this study may be linked to increased contact with nature in rural areas. However, research has also revealed the positive influence of nature-based interventions (as short as 3–4 days) on indicators of mental health and positive youth development in adolescents with little or no prior engagement with nature, including urban youth (Norton and Watt, 2014; Bowers et al., 2019; Parry et al., in press). Despite these opportunities, most young people across diverse contexts continue to report a high prevalence of sedentary behavior in time use studies (Arundell et al., 2016; Larson et al., 2019). Youth in urban communities, in particular, may face more pronounced barriers to outdoor recreation (Larson et al., 2011), including safety concerns (Veitch et al., 2006). Exploration and negotiation of barriers to nature-based experiences for youth across the rural-to-urban gradient could maximize potential developmental benefits. Additional research would help to reveal the longer-term implications of engagement with nature on young people's developmental growth, mental health, and stewardship behaviors in urban settings (Wray-Lake et al., 2019; Sachs et al., 2020).

In addition to consideration of place, the study was also limited in its consideration of intersectionality (Crenshaw, 1990; Godfrey and Burson, 2018). Most youth in our study attended schools in low-income communities, making inferences to higher-income populations difficult. Although the present study included race and gender as covariates, these potential dimensions of marginality should be more fully considered in future PYD work (Williams and Deutsch, 2016). Consistent with RDS metatheory, intersectional theory postulates that individual experiences of marginalized youth are influenced by multiple overlapping systems of injustice (Godfrey and Burson, 2018). Within rural communities, experiences can be quite heterogenous as marginalizing systems and the processes of class, race, gender, and culture meet (Cairns, 2013). Little consideration has been given for how these PYD processes may differ among diverse youth and across the rural to urban context (Paricio et al., 2020). Future work should explore whether the associations identified in the present study reflect commonality or specificity across different dimensions of diversity (Bornstein, 2017).

Despite these limitations, the present study provides additional support for link between connection to nature and PYD outcomes, reflecting an integration of the fields of recreation, conservation, and youth development. Findings extend the wealth of research on the psychological and developmental health benefits of nature; however, the study might also catalyze more interdisciplinary approaches to describing, explaining, and optimizing the lives of diverse young people (Baltes et al., 1977). An extensive literature has identified “developmental nutrients” provided by families, peers, schools, and afterschool programs (e.g., Benson et al., 2011). The present study adds to this work by examining nature as a resource for thriving.

### Implications for Practice

Youth time in nature and connection to the natural world are linked to healthy and positive developmental outcomes. When possible, parents and practitioners should therefore encourage youth from all backgrounds to engage with nature and participate in nature-based activities. These opportunities might not be accessible in all areas (e.g., urban neighborhoods), but the concept of “nearby nature” demonstrates how outdoor recreation opportunities can provide a variety of benefits close to home (Pyle, 2002; Wells and Evans, 2003; Chawla, 2015). Such an approach could work particularly well for youth in rural areas, where access to nature is often just beyond the doorstep. A “nearby nature” emphasis may be important for rural youth from low-income communities facing multiple adversities (Irvine et al., 2013), as nature can help youth become more resilient (Parry et al., in press). Increasing connections to nature for youth could be especially critical during times of high stress (Touloumakos and Barrable, 2020). For instance, youth who continued to engage in outdoor recreation reported better psychological outcomes during events such as the COVID-19 pandemic (Jackson et al., 2021). Maintaining access to nature-based recreation opportunities for youth is therefore critical, even more so during times of crisis (Ettekal and Agans, 2020).

Because connection to nature is closely linked to many PYD outcomes, youth program leaders might consider adopting an intentional approach to including more outdoor activities within their programming (Parry et al., 2021b). Our findings help make a case for nature as a viable setting for physical activity-based PYD programs. Although sport has gained a lot of traction for physical activity-based PYD (for a review, see Holt et al., 2017), the evidence base for nature-based programs is lacking (Parry et al., 2021a). By including measures of time in nature and connection to nature in program assessment (Frantz and Mayer, 2014), youth program leaders could explore and create new opportunities for positive development.

## Conclusion

Despite growing interest in the health and developmental benefits associated with youth time in nature (Louv, 2005), little research has examined the direct relationship between adolescents' nature-based experiences and holistic measures of positive youth development (PYD). Our study builds on previous efforts (Schusler and Krasny, 2010) by cultivating interdisciplinary discourse surrounding nature interaction and PYD. Our findings highlight the need for researchers and practitioners interested in PYD to consider integrating nature-related elements into their projects and programs. At a minimum, results suggest connection to nature might be viewed as a context-specific conceptualization of connection – a shift that would more closely align the five Cs with contemporary environmental issues and social movements that are important to many young people (Sachs et al., 2020). Ultimately, we propose that nature itself represents a valuable ecological asset that should be integrated into PYD frameworks.

## Data Availability Statement

The raw data supporting the conclusions of this article will be made available by the authors, without undue reservation.

## Ethics Statement

This project was reviewed and approved through the Institutional Review Board of Clemson University (IRB2014-429). Informed consent/assent was obtained from all individual participants.

## Author Contributions

EB and LL are the principal investigators of the study and oversaw conceptualization of the study, funding acquisition, and writing of the original draft. LL conducted data analysis and generated tables and figures. BP contributed to the literature review and reviewed and edited the manuscript. All authors contributed to the article and approved the submitted version.

## Conflict of Interest

The authors declare that the research was conducted in the absence of any commercial or financial relationships that could be construed as a potential conflict of interest.
