# Supplementary material for: Nature as an Ecological Asset for Positive Youth Development: Empirical Evidence From Rural Communities
Source: Front Psychol. 2021 Jun 4;12:688574. doi: 10.3389/fpsyg.2021.688574 (PMC8213347; doi:10.3389/fpsyg.2021.688574)
Supplement: Supplementary file 1 [file Data_Sheet_1.docx]

**Table S1**. Items (with descriptive statistics) used to measure two dimensions of the Nature-Relatedness Scale

| **Factor (with Items)** | **Mean** | ***SD*** |
| --- | --- | --- |
| **A. Nature-Relatedness (NR) - Experience**  **(3 items, Cronbach’s α = 0.866, McDonald’s ω = 0.868)** | **3.93** | **1.00** |
| My favorite places are outside in nature^a^ | 3.86 | 1.15 |
| I enjoy being outside in nature^a^ | 4.13 | 1.03 |
| I spend time outdoors whenever I can^a^ | 3.80 | 1.20 |
| **B. Nature-Relatedness (NR) - Self**  **(3 items, Cronbach’s α = 0.825, McDonald’s ω = 0.833)** | **3.53** | **1.00** |
| I feel very connected to all living things and the Earth^a^ | 3.53 | 1.18 |
| My relationship to nature is an important part of who I am^a^ | 3.57 | 1.18 |
| I think about how what I do affects the Earth^a^ | 3.53 | 1.12 |

^a^Item rated on scale: 1=Strongly disagree, 2=Disagree, 3=Neither, 4=Agree, 5=Strongly agree

**Table S2**. Items (with descriptive statistics) used to measure the Five Cs of Positive Youth Development (PYD)

| **Factor (with Items)** | **Theme/Reliability** | **Mean** | ***SD*** |
| --- | --- | --- | --- |
| **Overall PYD Scale (34 items)** | **Cronbach’s α = 0.880,**  **McDonald’s ω = 0.872** | **4.01** | **0.43** |
| **Competence (6 items)** | **Cronbach’s α = 0.740,**  **McDonald’s ω = 0.758** | **3.81** | **0.66** |
| I am just as smart as others my age.^a^ | Academic competence | 4.09 | 0.90 |
| I have a lot of friends.^a^ | Social competence | 4.26 | 0.93 |
| I could do well at just about any new athletic activity.^a^ | Physical competence | 3.68 | 1.13 |
| I do/did very well in my classwork at school.^a^ | Academic competence | 4.24 | 0.72 |
| I am better than others my age at sports.^a^ | Physical competence | 3.04 | 1.18 |
| I am popular with others my age.^a^ | Social competence | 3.68 | 1.05 |
| **Confidence (6 items)** | **Cronbach’s α = 0.837,**  **McDonald’s ω = 0.833** | **4.25** | **0.64** |
| I am happy with myself most of the time.^a^ | Self-worth confidence | 4.22 | 0.89 |
| I think I am good looking.^a^ | Appearance confidence | 3.82 | 1.06 |
| I really like the way I look.^a^ | Appearance confidence | 3.92 | 0.97 |
| I am happy being the way I am.^a^ | Self-worth confidence | 4.45 | 0.80 |
| All in all, I am glad I am me.^a^ | Positive identity confidence | 4.58 | 0.74 |
| When I am an adult, I’m sure I will have a good life.^a^ | Positive identity confidence | 4.47 | 0.73 |
| **Character (8 items)** | **Cronbach’s α = 0.646,**  **McDonald’s ω = 0.652** | **3.79** | **0.56** |
| Sometimes I do things I know I shouldn’t do. (Reverse-coded)^a^ | Conduct behavior character | 2.28 | 1.03 |
| I usually act the way I know I am supposed to.^a^ | Conduct behavior character | 4.01 | 0.86 |
| Helping to make the world a better place to live in.^b^ | Social conscience character | 4.22 | 1.02 |
| Giving time and money to make life better for other people.^b^ | Social conscience character | 4.02 | 1.01 |
| Doing what I believe is right even if my friends make fun of me.^b^ | Personal values character | 4.38 | 0.91 |
| Accepting responsibility for my actions when I make a mistake or get in trouble.^b^ | Personal values character | 4.32 | 0.94 |
| Knowing a lot about people of other races.^c^ | Values diversity character | 3.28 | 1.25 |
| Enjoying being with people who are of a different race than I am.^c^ | Values diversity character | 3.95 | 1.12 |
| **Caring (6 items)** | **Cronbach’s α = 0.833,**  **McDonald’s ω = 0.836** | **4.34** | **0.73** |
| When I see someone being taken advantage of, I want to help them.^d^ | Caring | 4.21 | 0.97 |
| It bothers me when bad things happen to any person.^d^ | Caring | 4.32 | 0.91 |
| I feel sorry for other people who don’t have what I have.^d^ | Caring | 4.36 | 0.92 |
| When I see someone being picked on, I feel sorry for them.^d^ | Caring | 4.49 | 0.87 |
| It makes me sad to see a person who doesn’t have friends.^d^ | Caring | 4.33 | 1.09 |
| When I see another person who is hurt or upset, I feel sorry for them.^d^ | Caring | 4.47 | 0.88 |
| **Connection (8 items)** | **Cronbach’s α = 0.802,**  **McDonald’s ω = 0.805** | **3.97** | **0.66** |
| I get a lot of encouragement at my school.^d^ | School connection | 3.84 | 1.00 |
| Teachers at school push me to be the best I can be.^d^ | School connection | 4.25 | 0.97 |
| I have lots of good conversations with my parents.^d^ | Family connection | 3.99 | 1.04 |
| In my family I feel useful and important.^d^ | Family connection | 4.21 | 1.02 |
| Adults in my town or city make me feel important.^d^ | Neighborhood connection | 3.70 | 1.05 |
| Adults in my town or city listen to what I have to say.^d^ | Neighborhood connection | 3.42 | 1.12 |
| I feel my friends are good friends.^e^ | Peer connection | 4.27 | 0.89 |
| My friends care about me.^e^ | Peer connection | 4.27 | 0.96 |

^a^Item rated on scale: 1=Strongly disagree, 2=Disagree, 3=Neither, 4=Agree, 5=Strongly agree

^b^Item rated on scale: 1=Not important, 2=Somewhat important, 3=Not sure, 4=Quite important, 5=Extremely important

^c^Item rated on scale: 1= Not at all like me, 2=A little like me, 3=Somewhat like me, 4=Quite like me, 5=Very much like me

^d^Item rated on scale: 1=Not well to 5=Very well

^e^Item rated on scale: 1=Almost never true or never true, 2=Seldom true, 3=Sometimes true, 4=Usually true, 5=Always true

**Table S3**. Items (with descriptive statistics) used to measure Contribution, the 6^th^ C of Positive Youth Development (PYD)

| **Factor (with Items)** | **Theme** | **Mean** | ***SD*** |
| --- | --- | --- | --- |
| **Overall Contribution (12 items)** | **Cronbach’s α = 0.733,**  **McDonald’s ω = 0.711** | **59.76^a^** | **15.21** |
| I often think about doing things so that people in the future can have things better.^b^ | Ideology | 3.78 | 0.99 |
| It is important to me to contribute to my community and society.^b^ | Ideology | 3.88 | 0.94 |
| It’s not really my problem if my neighbors are in trouble and need help. (Reverse-coded)^b^ | Ideology | 3.92 | 1.13 |
| If I had to choose between helping to raise money for a neighborhood project and enjoying my own free time, I’d keep my freedom. (Reverse-coded)^b^ | Ideology | 3.24 | 1.21 |
| Think about how you see your future. What are your chances for the following: Be involved in community service^c^ | Ideology | 3.60 | 1.15 |
| Think about how you see your future. What are your chances for the following: Be involved helping other people^c^ | Ideology | 4.06 | 1.03 |
| “Helping” includes any activity that you are not required to do but you do to improve things or make things easier for other people. How often do you do the following things:  Help a friend^d^ | Time helping | 3.35 | 0.76 |
| “Helping” includes any activity that you are not required to do but you do to improve things or make things easier for other people. How often do you do the following things:  Help a neighbor^d^ | Time helping | 2.45 | 1.10 |
| During the last 12 months, how many times have you been a leader in a group or organization?^e^ | Leadership | 2.41 | 1.41 |
| We want to know how often you participate in the following community clubs or activities during the year: School government^f^ | Service activity participation | 0.61 | 1.33 |
| We want to know how often you participate in the following community clubs or activities during the year: Mentoring other students/Peer advising^f^ | Service activity participation | 0.87 | 1.47 |
| We want to know how often you participate in the following community clubs or activities during the year: Volunteering your time^f^ | Service activity participation | 1.82 | 1.64 |

^a^Contribution scale score was created by recoding all items on a scale of 0-5, weighting the time helping, leadership and service activity participation categories equally to create a “Contribution Action” score, then weighting this score equally with the four items used to create a “Contribution Ideology” score. The final score was the rescaled to range from 0-100.

^b^Item rated on scale: 1=Strongly disagree, 2=Disagree, 3=Neither, 4=Agree, 5=Strongly agree

^c^Item rated on scale: 1=Very low, 2=Low, 3=About 50/50, 4=High, 5=Very high

^d^Item rated on scale: 0=Never, 1=Seldom, 2=Sometimes, 3=Often, 4=Very often

^e^Item rated on scale: 0=Never, 1=Once, 2=Twice, 3=3-4 times, 4=5 or more times

^f^Item rated on scale: 0=Never, 1=Once a month or less, 2=A couple of times a month, 3=Once a week, 4=A few times a week, 5=Every day

**Table S4.** Parameter estimates in OLS regression model examining association between time in nature, connection to nature, and overall PYD scale scores in a sample of rural middle school students in South Carolina, USA, controlling for demographic variables (n = 587).

|  |  | **Overall PYD^a^** | |
| --- | --- | --- | --- |
|  |  | (*M*=4.03) | |
| **Variable** | **Mean** | **B** | **SE** |
| TimeOutdoors^b^ | 1.69 | 0.001 | 0.01 |
| NR-Experience^c^ | 3.93 | 0.042* | 0.02 |
| NR-Self^c^ | 3.53 | 0.130*** | 0.02 |
| INS^c^ | 3.39 | 0.031* | 0.02 |
| Age^d^ | 12.91 | -0.038 | 0.02 |
| Gender (female) | 0.56 | 0.014 | 0.04 |
| Race (white) | 0.60 | -0.042 | 0.04 |
| Model Adj. *R*^2^ |  | 0.189 | |

*,**,*** denote statistical significance at α = 0.10, 0.05, and 0.01, respectively

^a^PYD scales scores ranged from 1 to 5, with higher scores representing higher levels of PYD

^b^TimeOutdoors refers “time outdoors in nature,” recoded as continuous scale based on the following categories: none (0 hr), less than 0.5 hr per day (0.25 hr), between 0.5 and 1 hr per day (0.75 hr), between 1 and 2 hr per day (1.5 hrs), between 2 and 3 hr per day (2.5 hrs), between 3 and 4 hr per day (3.5 hrs), between 4 and 5 hr per day (4.5 hrs), more than 5 hr per day (5.5 hrs).

^c^Scores for nature relatedness (NR) scales (both experience and self) and the inclusion of nature in self (INS) scale ranged from low connection (1) to strong connection (5)

^d^Student ages ranged from 11 to 14 years old.

**Table S5.** Parameter estimates in OLS regression models examining associations between time in nature, connection to nature, and different dimensions of PYD (Five Cs, plus contribution) in a sample of rural middle school students in South Carolina, USA, controlling for demographic variables (n = 587).

|  | **Competence^a^** | | **Confidence^a^** | | **Character^a^** | | **Connection^a^** | | **Caring^a^** | | **Contribution^b^** | |
| --- | --- | --- | --- | --- | --- | --- | --- | --- | --- | --- | --- | --- |
|  | (*M*=3.82) | | (*M*=4.26) | | (*M*=3.80) | | (*M*=3.98) | | (*M*=4.35) | | (*M*=59.87) | |
| **Variable** | **B** | **SE** | **B** | **SE** | **B** | **SE** | **B** | **SE** | **B** | **SE** | **B** | **SE** |
| TimeOutdoors^c^ | 0.057*** | 0.02 | 0.007 | 0.02 | -0.011 | 0.02 | 0.006 | 0.02 | -0.049** | 0.02 | -0.572 |  |
| NR-experience^d^ | 0.134*** | 0.04 | 0.039 | 0.04 | -0.031 | 0.03 | 0.040 | 0.04 | 0.049 | 0.04 | 0.859 | 0.83 |
| NR-self^d^ | 0.004 | 0.04 | 0.081** | 0.04 | 0.206*** | 0.03 | 0.107*** | 0.04 | 0.237*** | 0.04 | 4.580*** | 0.080 |
| INS^d^ | 0.064** | 0.03 | 0.03 | 0.03 | 0.021 | 0.03 | 0.058** | 0.03 | -0.013 | 0.03 | 1.608** | 0.64 |
| Age^e^ | -0.011 | 0.04 | -0.32 | 0.04 | -0.012 | 0.03 | -0.064* | 0.04 | -0.079* | 0.04 | 0.086 | 0.83 |
| Gender (female) | -0.065 | 0.06 | -0.084 | 0.06 | 0.080 | 0.05 | 0.021 | 0.06 | 0.099 | 0.06 | 3.669*** | 1.24 |
| Race (white) | -0.062 | 0.06 | -0.279*** | 0.06 | -0.023 | 0.05 | 0.104* | 0.06 | 0.002 | 0.06 | -1.368 | 1.25 |
| Model Adj. *R*^2^ | 0.143 | | 0.074 | | 0.114 | | 0.104 | | 0.123 | | 0.162 | |

*,**,*** denote statistical significance at α = 0.10, 0.05, and 0.01, respectively

^a^PYD scales scores ranged from 1 to 5, with higher scores representing higher levels of each dimension of PYD

^b^Contribution scores ranged from 1 to 100, with higher scores representing higher levels of contribution

^c^TimeOutdoors refers “time outdoors in nature,” recoded as continuous scale based on the following categories: none (0 hr), less than 0.5 hr per day (0.25 hr), between 0.5 and 1 hr per day (0.75 hr), between 1 and 2 hr per day (1.5 hrs), between 2 and 3 hr per day (2.5 hrs), between 3 and 4 hr per day (3.5 hrs), between 4 and 5 hr per day (4.5 hrs), more than 5 hr per day (5.5 hrs).

^d^Scores for nature relatedness (NR) scales (both experience and self) and the inclusion of nature in self (INS) scale ranged from low connection (1) to strong connection (5)

^e^Student ages ranged from 11 to 14 years
